# Efficient Temporal Coding in the Early Visual System: Existing Evidence and Future Directions

**DOI:** 10.3389/fncom.2022.929348

**Published:** 2022-07-04

**Authors:** Byron H. Price, Jeffrey P. Gavornik

**Affiliations:** Center for Systems Neuroscience, Graduate Program in Neuroscience, Department of Biology, Boston University, Boston, MA, United States

**Keywords:** efficient coding, predictive coding, time, temporal representations, visual cortex

## Abstract

While it is universally accepted that the brain makes predictions, there is little agreement about how this is accomplished and under which conditions. Accurate prediction requires neural circuits to learn and store spatiotemporal patterns observed in the natural environment, but it is not obvious how such information should be stored, or encoded. Information theory provides a mathematical formalism that can be used to measure the efficiency and utility of different coding schemes for data transfer and storage. This theory shows that codes become efficient when they remove predictable, redundant spatial and temporal information. Efficient coding has been used to understand retinal computations and may also be relevant to understanding more complicated temporal processing in visual cortex. However, the literature on efficient coding in cortex is varied and can be confusing since the same terms are used to mean different things in different experimental and theoretical contexts. In this work, we attempt to provide a clear summary of the theoretical relationship between efficient coding and temporal prediction, and review evidence that efficient coding principles explain computations in the retina. We then apply the same framework to computations occurring in early visuocortical areas, arguing that data from rodents is largely consistent with the predictions of this model. Finally, we review and respond to criticisms of efficient coding and suggest ways that this theory might be used to design future experiments, with particular focus on understanding the extent to which neural circuits make predictions from efficient representations of environmental statistics.

## Introduction

An interesting feature of the human mind is how it tricks us into thinking complex tasks are simple. One semi-apocryphal illustration of this was Marvin Minsky asking an undergraduate to program a computer to “describe what it saw” through a camera, over the course of a single summer. Minsky’s wild underestimate of how hard this would be stemmed from an intuition that it can’t be terribly hard to do something so effortless. Similarly, our innate understanding of time as the inescapable dimension along which life proceeds seems effortless. Surely, the mechanistic underpinnings of this ability should be easy to describe? Not quite. It is not even easy to define what “time” is. Though multiple research groups have made important theoretical and experimental contributions to understanding time in the brain (Mauk and Buonomano, 2004; Buonomano and Laje, 2010; Allman et al., 2014; Eichenbaum, 2014; Merchant et al., 2014, 2015; Tucci et al., 2014; Finnerty et al., 2015; Petter et al., 2018; Wang et al., 2018; Balasubramaniam et al., 2021), neuroscience provides few satisfying answers to the big questions of how time is explicitly represented in cortex, how temporal relationships are stored in memories, and how memories of temporal relationships are used to make predictions.

The ability to make accurate predictions confers clear competitive advantages. Accurately extrapolating the trajectory of a moving object to predict its future state, for example, is very useful for both prey capture and predator evasion. An interesting idea is that prediction might emerge as a natural consequence of resource optimization, thus providing dual benefits for adaptive behavior and energy efficiency. The concept of *efficient coding* from information theory describes how to achieve such resource optimization for data storage or transmission (Shannon, 1948; Cover and Thomas, 2006b; Stone, 2018). When data are extended in space or time, efficient coding suggests that a *predictive coding* scheme can be used to compress information and save energy (Elias, 1955). Though these ideas were inspired by problems in telecommunications engineering, they have found significant application in biology.

Efficient coding was first introduced to neuroscience by Attneave (1954) and Barlow (1961), who argued that retinal circuits use efficient coding to transform light patterns into the neural code transmitted through the optic nerve. The basic concept has evolved through the years and been used to explain a wide variety of experimental results across the visual system (Srinivasan et al., 1982; Atick and Redlich, 1992; Dong and Atick, 1995; Dan et al., 1996; Olshausen and Field, 1996; Meister and Berry, 1999; Doi et al., 2012; Pitkow and Meister, 2012). Many basic functional properties of the retina are likely the result of efficient coding, for example the unequal distribution of ON and OFF ganglion cell types (Balasubramanian and Sterling, 2009). Srinivasan et al. (1982) were the first to show that retinal ganglion cells (RGCs) effectively act as linear predictive coders. Extending this notion, Ocko et al. (2018) provided a plausible explanation for the existence of multiple ganglion cell subtypes. Efficient coding may also provide a framework to explain how the evolution and plasticity of cortical circuitry is shaped by natural environmental statistics (Olshausen and Field, 1996; Bell and Sejnowski, 1997; Rao and Ballard, 1999; Wiskott and Sejnowski, 2002; Jehee et al., 2006; Creutzig and Sprekeler, 2008). For example, Olshausen and Field (1996, 1997) created a spatial efficient coding model that mimics primary visual cortex (V1) receptive fields when trained on natural scenes. Rao and Ballard (1999) famously introduced a hierarchical predictive coding model to explain the classical and extra-classical receptive field properties of V1 neurons. Related models have since been suggested to provide a general framework for understanding cortical function (Friston, 2005; Bastos et al., 2012; Spratling, 2017; Whittington and Bogacz, 2019; Millidge et al., 2021).

Overall, there is a rich literature on both efficient and predictive coding. In the visual system, however, much of the experimental and theoretical focus has been in the spatial domain. This may reflect the inherent “spatial-ness” of vision as a sensory modality, but we argue that time is also fundamental, even beyond motion processing. In addition, the notion of predictive coding has become somewhat restrictive in its potential instantiations in neural circuitry, e.g., prediction and predictive coding may still be relevant to understanding cortical function even if hierarchical predictive coding is the inappropriate model. To that end, this review begins with a primer on efficient coding wherein we explicitly derive the close relationship between efficient and predictive coding. Next, we review evidence for efficient coding in the retina and dorsal lateral geniculate nucleus (dLGN). We then move to later visual areas, with particular emphasis on how efficient coding principles in visual cortex may underlie a variety of time-dependent computational tasks, including visual flow processing, spatiotemporal sequence learning, and adaptation. In the end, we hope to clarify the sometimes confusing relationship between efficient and predictive coding, and discuss ways in which these theories may guide experiments and provide clues about how the nervous system codes temporal relationships to make predictions.

## Efficient Coding Primer

Efficient coding is a concept from information theory describing how data can be transmitted or stored with minimal use of energy, time, and resources. Pioneered in the 1920s–1950s by Harry Nyquist, Ralph Hartley, and Claude Shannon of Bell Telephone Labs, information theory provided a quantitative framework to analyze then emergent telecommunications technology and help their employer save money on telegram and telephone transmission. The goal was to design a system capable of reliable message transmission and storage.

As a concrete example, consider constructing a message from a four-letter alphabet, θ = {*A, B, C, D*}. There are 4^10^ possible 10-letter messages, a typical example of which might be BACAAABDAA. If we assume that each letter appears independently within a message according to the following probabilities:


$$P\left(A\right)=\frac{1}{2},P\left(B\right)=\frac{1}{4},P\left(C\right)=\frac{1}{6},P\left(D\right)=\frac{1}{12}$$

then certain messages are much more likely to occur than others (DDDDDDDDDD is very unlikely, for example). It makes intuitive sense to choose an *encoding scheme* that takes advantage of this non-uniformity. With this in mind, Shannon introduced the idea of *entropy* to quantify the average number of symbols required to store or send any such message (Shannon, 1948; Cover and Thomas, 2006c). In his formulation, information is simply *I* (*X*) = −log_2_ (*P*(*X*)) bits, which is the number of binary digits required to store a message that occurs with probability *P* (*X*). Due to its inverse relationship with probability, information is a measure of epistemic surprise relative to expectation, and entropy is just the expectation of the information:


H(θ)≜E[I(X)]

The average total amount of information in a message of *N* symbols is simply NH(θ). In our example,


$$H\left(\theta \right)=-\sum_{x\in \left\{A,B,C,D\right\}}P\left(X=x\right){\text{log}}_{2}\left(P\left(X=x\right)\right)=1.73\text{bits}$$

Shannon’s *source coding theorem* proves that the entropy defines the minimum possible number of bits that can be used to represent one symbol from our messages without losing information. For this value of entropy, the average information in a 10-symbol message would be 17.3 bits. If we had assumed that all letters were equally likely, then entropy would be 2 bits and the average 10-symbol message would be 20 bits. As this example demonstrates, the source coding theorem implies that data from different distributions can be stored with differing amounts of information. The question is how to design an encoding scheme that takes advantage of this theorem, and minimizes the number of bits utilized.

According to the source coding theorem, an encoding scheme is efficient if messages are, on average, transmitted with a number of bits approaching the entropy of the source (Shannon, 1948; Barlow, 1961; Atick, 1992; Cover and Thomas, 2006a; Sterling and Laughlin, 2015). Efficient codes specify messages using the minimum possible number of bits and do so by removing predictable information.

To get a sense of what this means in practice, consider representing our symbol alphabet using a simple binary encoding scheme:

**Table:** 

A	00
B	01
C	10
D	11
	

Transmitting a letter/symbol with this scheme requires 2 bits, which is more than the theoretical minimum of 1.73 bits implied by the source coding theorem. What exactly makes this code inefficient? The answer is redundancy: messages transmitted with this code are, on average, predictable. To see this, consider transmitting a 0 for the first bit. Since $P\left(A\right)=\frac{1}{2}$ and $P\left(B\right)=\frac{1}{4}$, the probability that the second bit will also be a zero is 2/3. A more efficient code would reduce such redundancies.

Formally, Shannon redundancy is given by Atick (1992):


$$R=1-\frac{H\left(\theta \right)}{C}$$

where *C = 2* bits is the average message length in our encoding scheme. The redundancy is always between 0 and 1, with a perfectly efficient code having *H* (θ) = *C* and *R = 0*. In this case, *C = 2* and *H* (θ) = 1.73, so *R* = 0.135.

Consider an alternative 3-bit encoding scheme:

**Table:** 

A	0
B	10
C	110
D	111
	

Intuition might suggest that adding an extra bit will decrease efficiency, but this is incorrect when we consider the underlying message-generating process. While C and D both require 3 bits to transmit, this relative increase in message length might be compensated by the fact that the most common symbol, A, requires only 1 bit. For the entire 3-bit scheme:


C=P(A)×(1bit)+P(B)×(2bits)+(P(C)+P(D))×(3bits)=1.75bits

The redundancy is now *R* = 0.011, an order of magnitude smaller than the 2-bit scheme. As long as the statistics remain stationary, this 3-bit scheme represents a very efficient code to transmit our messages and illustrates the fundamental principle of efficient coding: *use relatively fewer symbols to encode prevalent/expected messages and relatively more symbols to encode rare/unexpected messages* (Cover and Thomas, 2006a).

Our example also demonstrates the principle that deviations from uniformity *decrease* entropy (1.73 bits is less than the 2 bits of a uniform distribution). As more complex statistical dependences are added to the source, entropy often drops even more. Consider for example a Markov chain with the following transition probability matrix, *T*:

**Table:** 

	A	B	C	D
A	0.1	0.7	0.1	0.1
B	0.1	0.1	0.7	0.1
C	0.1	0.1	0.1	0.7
D	0.7	0.1	0.1	0.1
				

The transition matrix provides conditional probabilities such as *P* (*B* | *A*) = 0.7. This Markov chain generates sequences that tend to repeat the pattern ABCD, so neighboring message elements are no longer statistically independent (*P* (*AB*) ≠ *P* (*A*) *P* (*B*)). According to the mathematics of Markov chains, the asymptotic probability of element occurrence, π, solves the equation π = π*T*. Thus, π is the left eigenvector of the transition matrix, whose corresponding eigenvalue is 1. In this case,


$$\pi \left(X\right)=\left\{ \begin{array}{c} 0.25,X=A\\ 0.25,X=B\\ 0.25,X=C\\ 0.25,X=D \end{array}\right.$$

This is known as the stationary distribution of the Markov chain, representing how often we expect each symbol to occur regardless of its position within the message. For Markov chains, the so-called *entropy rate* becomes (Cover and Thomas, 2006d):


$$H\left(\theta \right)≜-\sum_{x\in \left\{A,B,C,D\right\}}\pi \left(X=x\right)\sum_{y\in \left\{A,B,C,D\right\}}P\left(Y=y|X=x\right){\text{log}}_{2}\left(P\left(Y=y|X=x\right)\right)$$

For the present example:


H(θ)=1.357bits

This represents a significant reduction in entropy compared to the 2 bits of a 4-symbol uniform distribution, brought about by the statistical dependence between neighboring message elements. *A* is now predictive of *B*, and *B* of *C*, etc. despite each letter being equally likely overall.

An efficient code for this Markov distribution might look like this:

**Table:** 

	A	B	C	D
A	10	0	110	111
B	111	10	0	110
C	110	111	10	0
D	0	110	111	10
				

If the previous symbol was an A and we receive a 0, the encoding matrix tells us that the second symbol must be *B*. After that, we get another *0*, so *C*. Here is an example sequence generated by this Markov process and its corresponding binary representation (assuming all messages start with A):


$$\begin{array}{c} \left(\text{A}\right)\mathrm{BCBCDBABCCDCDA}\\ 00111001101110010011100 \end{array}$$

In the absence of noise, we can perfectly reconstruct the original message from its binary counterpart. The average, long-run per-symbol message length is:


C=P(A|A)×2+P(B|A)×1+P(C|A)×3+P(D|A)×3=1.5bits

**FIGURE 1:**
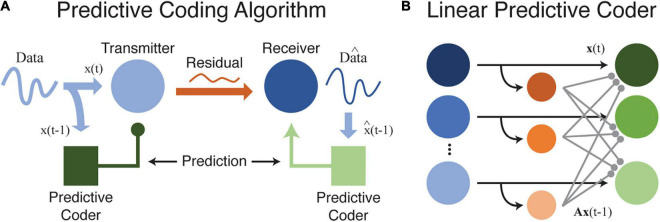
**(A)** This cartoon illustrates how data can be efficiently transferred between a source and receiver by using a predictive coder to first remove and then recover redundant information. Ideally, and in the absence of internal noise, data transmitted between source and receiver is fully compressed with no redundancy or predictability. While this diagram is based on efficient coding theory as formalized for data transfer and storage in telecommunication systems, the same principles may apply to neural circuits as well. **(B)** A simple model illustrating how a predictive coder could be implemented by a neural circuit. Output neurons (green) receive excitatory inputs and a delayed inhibitory input transformed by a weight matrix A (orange-to-green connections). Neurons at the output transmit the residual difference between the current input, x_*t*_, and the predicted input, Ax_(_*_*t*_*_–1)_.

Redundancy is therefore *R* = 0.096. There is still some room for improvement, but this *predictive coding* scheme is much more efficient than schemes that ignore the statistical dependence between neighboring elements (Elias, 1955; Huang and Rao, 2011; Spratling, 2017). As before, an efficient code uses more information to represent unexpected events, but inclusion of an ordinal (or temporal) relationship changes our interpretation. Now, our efficient code uses more information (more bits) to represent *prediction errors* relative to expectation: when *D* comes after *C*, we expect that and use only 1 bit. We do not expect *A* to follow *C* and use correspondingly more bits (3) when this occurs. This is precisely the predictive coding algorithm as originally proposed for efficient transmission of telecommunications data (Elias, 1955). Figure 1 shows how predictive coding might be used to efficiently transfer information, including in a neural environment.

To conclude, it is worth noting that efficient coding is perfectly valid in the presence of internal noise, such as when bits randomly flip during transmission. If the original encoding scheme is optimal but bits flip randomly during transmission, then information is lost at the receiving end. This is no longer an efficient code by definition, so to account for noise, efficient codes ultimately represent a trade-off between minimizing the average message length and preserving redundancy to be robust to noise (Atick, 1992; Atick and Redlich, 1992; van Hateren, 1992; Dong and Atick, 1995; Simoncelli, 2003). Efficient coding is therefore typically formalized as a maximization of mutual information between sensory inputs and neural responses, rather than a minimization of redundancy (Doi et al., 2012). This encourages neural codes that have low redundancy, but also high discriminability of different stimuli with low trial-to-trial variability for the same stimulus. Many authors include an additional energy or metabolic constraint to encourage some form of sparseness in the ultimate solution (Olshausen and Field, 1996; Chalk et al., 2018; Ocko et al., 2018).

### Lessons for Predictive Coding in the Nervous System

Lessons from this primer relevant to understanding temporal processing in the nervous system are: (1) Predictive coding is a direct consequence of efficient coding, particularly when applied to sequential or autocorrelated data. (2) A code that is efficient for one statistical distribution is inefficient for other distributions, implying that neural codes should be optimized for the natural environment and ought to adapt to changing environmental statistics to maintain efficiency. (3) To efficiently encode sequential data, it is necessary to learn sequence order and, if extended into the temporal domain, timing as well. Prediction emerges naturally as a consequence of efficient coding without requiring a separate representational framework.

The theory suggests that significant energy savings are possible. To achieve this, however, requires knowledge of the distribution of sensory inputs, ideally even the joint distribution of inputs and motor outputs. But, the process of learning the relevant distributions often falls beyond the purview of traditional efficient coding theory. Neural structures supporting efficient coding can, in principle, develop generationally through evolution (Zador, 2019) or within a lifetime through unsupervised or self-supervised learning algorithms (Oja, 2002; Hosoya et al., 2005; van den Oord et al., 2018; Bakhtiari et al., 2021; Zhuang et al., 2021). It should be noted that there is some terminological ambiguity around the phrase “predictive coding,” which often refers to specific unsupervised learning algorithms, premised on various assumptions about neural structure and function (Rao and Ballard, 1999; Friston, 2005; Huang and Rao, 2011; Spratling, 2017; Whittington and Bogacz, 2019; Grossberg, 2021; Millidge et al., 2021). These do not necessarily comport with Shannon’s efficient coding formalism. Further adding to the ambiguity, predictive processing is often described as a general principle of cortical function, encompassing development, learning, and efficient neural encoding (Wiskott and Sejnowski, 2002; Bialek et al., 2007; Palmer et al., 2015; Keller and Mrsic-Flogel, 2018; Singer et al., 2018; Bakhtiari et al., 2021). Here, we generally refer to predictive coding as a way to encode and compress data from certain distributions, rather than as a learning algorithm. To see how efficient coding relates to nervous system function, we now summarize how the concept has served our understanding of the retina.

## Efficient Coding in Retina and Thalamus

From an information-theoretic perspective, neural coding in retinal photoreceptors is inefficient because the statistics of natural visual scenes create activity patterns that are highly correlated in space and time. Any code that simply recapitulates this structure would be inefficient in space, energy, and resource utilization (Landauer, 1976; Balasubramanian and Sterling, 2009; Sterling and Laughlin, 2015). Such inefficiency would then be exacerbated by spiking RGCs, because spikes are particularly expensive in terms of energy consumption, and higher average firing rates require increasingly greater axonal volumes (Laughlin et al., 1998; Attwell and Laughlin, 2001; Balasubramanian et al., 2001; Laughlin, 2001; Perge et al., 2009). Space leaving the retina is very limited (in humans, there are two orders of magnitude fewer axons in the optic nerve, 10^6^, than there are photoreceptors, 10^8^), so a more efficient representation is advantageous. Attneave (1954) and Barlow (1961, 1989) were the first to recognize this and apply Shannon’s theory to neuroscience. Barlow proposed that RGCs encode and transmit visual information using a simple efficient coding heuristic: reduce redundancy by generating fewer action potentials for expected visual inputs and more spikes for unexpected ones. The efficient code is actually created *via* filtering operations in retinal circuits, which throttle firing rates and decrease redundancy by removing many of the input correlations imparted by natural scene statistics (Atick and Redlich, 1992; Meister and Berry, 1999; Pitkow and Meister, 2012).

Barlow’s hypothesis appears to be approximately correct (see Meister and Berry, 1999; Balasubramanian and Sterling, 2009; Huang and Rao, 2011 for reviews). RGCs are effectively linear predictive coders (Elias, 1955; Srinivasan et al., 1982; Meister and Berry, 1999; Huang and Rao, 2011; Doi et al., 2012) with receptive fields resembling those predicted by efficient coding models (Atick and Redlich, 1992; Doi et al., 2012; Ocko et al., 2018). In the temporal domain, linear RGC receptive field filters are biphasic and compute the difference between recent past and present. Spatially, RGCs have a difference-of-Gaussians organization that compares luminance between center and surround regions. In both cases, RGCs can be understood to predict correlations, responding minimally when they are present (e.g., when luminance patterns are constant in time or uniform across the spatial extent of the receptive field) and responding maximally to expectation violations (e.g., when luminance changes rapidly in space or time). These retinal filters have even been shown to adapt over rapid timescales to more efficiently encode visual information from novel distributions (Hosoya et al., 2005). Beyond the retina, relay neurons in the dLGN continue this process, especially in the temporal domain (Saul and Humphrey, 1990; Hartveit, 1992; Dong and Atick, 1995; Dan et al., 1996) where whitening occurs in a manner consistent with the efficient coding hypothesis (Dong and Atick, 1995; Dan et al., 1996). For example, the efficient coding model of Dong and Atick explains the existence of lagged and non-lagged dLGN relay neurons, as observed in physiological data (Hartveit, 1992).

Despite this evidence, it is not clear that the same principles are sufficient to explain the early visual system in all its complexity. One common critique is that information theory derives from the general principle that all information is created equal. In the real world, some sources of information are more relevant to an organism than others. Frogs, for example, are better served by a visual system evolutionarily tuned to detect and locate flies than by an abstract requirement to efficiently compress all visual information equally.

Several groups have proposed variations on efficient coding that call for more nuanced perspectives by taking messy biological imperatives and constraints into account. Balasubramanian and Sterling (2009) and Sterling and Laughlin (2015), for example, argue that it is advantageous to minimize per-bit computational costs, even while acknowledging that some information sources may be relatively privileged due to their ethological importance. These researchers and others have gone to great lengths to measure the computational cost of information transmission in terms of quantities like axonal volume and ATP consumption. Their more holistic approach leads to a variety of predictions regarding the structure and function of the retina that are well-supported by empirical data (Borghuis et al., 2008; Balasubramanian and Sterling, 2009; Perge et al., 2009; Gjorgjieva et al., 2014).

Another variation, proposed by Zhao et al. (2012); Teulière et al. (2015), Lelais et al. (2019), and Eckmann et al. (2020) takes the name of *active efficient coding* and is built around an interesting observation: since environmental statistics are partially governed by the animal’s own behavior, changing behavior can make a neural code more or less efficient. This idea leads to a variety of empirical predictions regarding the relationship between sensation and action. Teulière et al. (2015) have shown, for example, that both vergence and smooth pursuit eye movements can be learned *de novo* in artificial systems that optimize coding efficiency by simultaneously adjusting both the neural representation and eye movements (Zhao et al., 2012; Lelais et al., 2019).

Finally, and particularly relevant to our focus on time, is a hypothesis proposed by Bialek et al. (2007). Efficient codes compress data down to Shannon’s source coding limit. Beyond that limit, rate-distortion theory provides a principled way to select certain information for deletion. Inspired by this idea and a related framework known as the information bottleneck (Tishby et al., 2000), Bialek et al. suggested that sensory systems preferentially delete information about the past. They reasoned that predictive information (about the future) is uniquely useful for action and decision-making, and should therefore be prioritized. Such predictive information, which is inconsistent with traditional models of visual processing, has been observed in the early visual system of various species (Palmer et al., 2015; Salisbury and Palmer, 2015; Sachdeva et al., 2021; Wang et al., 2021).

While the retina and dLGN provide some of the best support for the idea that neural circuits are shaped by environmental statistics to efficiently encode information, there is evidence that similar considerations may hold in other visual areas as well.

## Efficient Coding in Primary Visual Cortex

Information sources that exhibit statistical dependences across space and time can be compressed by efficient encoding schemes. In the visual system, part of this process occurs in the retina and dLGN, which perform spatial and temporal decorrelation relative to the statistical structure of natural visual inputs (Srinivasan et al., 1982; Dong and Atick, 1995; Dan et al., 1996; Hosoya et al., 2005; Huang and Rao, 2011; Pitkow and Meister, 2012). However, temporal decorrelation appears limited to brief timescales, on the order of ∼30–300 ms, and processing is largely linear, subtracting the mean and removing pairwise correlations (though see, for example, Palmer et al., 2015 for more complex processing in the retina). Longer-timescale and higher-order correlations present in natural visual inputs survive the initial processing stages. This suggests that information reaching V1 is still inefficiently encoded. Regardless of the ultimate computational goal or task, an inefficient representation is in general more energetically costly and more difficult for downstream regions to process, as argued by Barlow (1990). V1 may therefore construct a more efficient representation, acting on longer timescales and reducing higher-order correlations. Examples of higher-order correlations in natural vision are edges in the spatial domain (representing correlations between spatially adjacent center-surround receptive fields) and brief trajectories in the temporal domain [which V1 can rapidly learn to predict (Xu et al., 2012)]. Canonical V1 receptive fields can be understood to operate on both forms of correlation in an efficient coding sense. “Edge detectors” in V1 eliminate spatial correlations found in natural scenes by integrating information across multiple dLGN inputs (Olshausen and Field, 1996; Bell and Sejnowski, 1997). V1 neurons with “space-time inseparable,” or direction-selective receptive fields, similarly eliminate higher-order correlations associated with motion trajectories.

Predictive coding models consistent with efficient coding have been used to explain both classical and extra-classical receptive field properties of V1 neurons, especially in the spatial domain (Spratling, 2010; Huang and Rao, 2011; Keller and Mrsic-Flogel, 2018). The well-known Rao and Ballard (1999) hierarchical predictive coding model learns V1-like receptive fields when trained on natural scenes, showing both classical Gabor-like spatial structure and extra-classical effects like end stopping (neural firing evoked by elongated bars increases with bar length up to some critical length, beyond which firing rapidly decreases). Cells with this property were originally called hypercomplex by Hubel and Wiesel (1965), but are now termed end stopped (Gilbert, 1977). Such *contextual effects* are often consistent with efficient coding models but difficult to reconcile with strictly feedforward models of visual processing (Schwartz et al., 2007; Huang and Rao, 2011; Carandini and Heeger, 2012; but see Priebe and Ferster, 2012 for an alternative explanation of some contextual effects).

In the following sections, we review evidence for temporal efficient coding in V1, especially focusing on data from rodents.

### Visual Flow

Visual flow caused by self-motion is responsible for large amounts of neural activity in the early visual system. Given the canonical properties of neurons in V1 (acting as edge detectors … responding to increments or decrements of light but not generally to steady-state luminance sources … showing direction selectivity), an animal’s natural motion through the environment, along with associated body and head-orienting movements, ought to evoke significant firing. In addition to the purely visual information evoked by such behaviors, body movements associated with locomotion and head orienting also evoke activity in rodent V1 (Vinck et al., 2015; Stringer et al., 2019; Guitchounts et al., 2020; Parker et al., 2022). This activity persists even in the dark, and may represent corollary discharge signals from motor areas or perhaps even predictions of the sensory consequences of movement (Lappe et al., 1999; Shadmehr et al., 2010; Leinweber et al., 2017; Sawtell, 2017). The predictable spatiotemporal correlations created by visual flow, head movements, and locomotion therefore make these strong candidates for efficient predictive coding.

**FIGURE 2:**
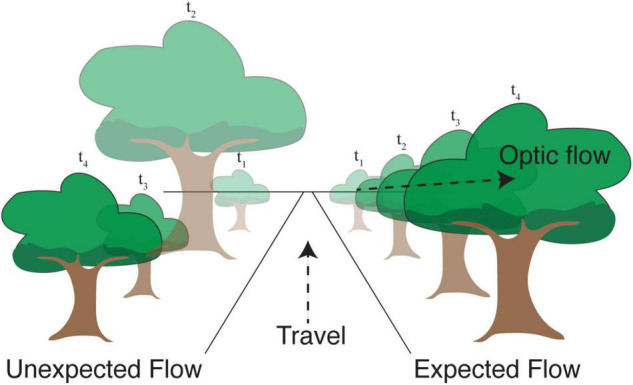
Movement in the direction shown causes objects in the visual field to progress along predictable trajectories. Right: A tree that is in the distance at time *t*_1_ becomes larger and moves toward the right at later time points. Efficient coding suggests that the visual response resulting from this expected apparent motion in visual space ought to be relatively small. Left: If this progression were scrambled in time, the resulting “unexpected optic flow” would cause the same visual images to produce relatively larger neural responses signifying an expectation violation.

There are at least two different ways in which efficient coding may shape cortical responses to natural visual flow. The first is by forming compressed representations of flow-like inputs by eliminating statistically predictable dependences between neighboring moments in time (as in our example discussing efficient encoding of Markov chains). Second, though related, external motion relative to the animal creates an unexpected visual input with respect to locally predicted visual flow. In both cases, efficient representations would be expected to generate relatively larger responses when unexpected flow patterns violate spatiotemporal predictions (Figure 2). Even if the system does not take advantage of such a mechanism to conserve energy, it could still benefit from knowing the distribution of flow signals, which necessarily involves some ability to predict the future.

A series of papers by Keller and colleagues has extensively studied visual flow in the context of predictive coding (Keller et al., 2012; Zmarz and Keller, 2016; Attinger et al., 2017; Leinweber et al., 2017; Jordan and Keller, 2020). They first looked at dense, topographically organized projections from secondary motor cortex to V1 layer 2/3, finding functional evidence for corollary discharge and visual flow feedback transmitted to V1 (Leinweber et al., 2017). They also discovered *mismatch receptive fields* in V1, which seemed to signal prediction errors relative to the expected visual flow: about 10–20% of recorded neurons were tuned to the properties of visual flow perturbations, for example video playback halts during active, coupled locomotion (Zmarz and Keller, 2016). In the latter study, mice were trained to navigate a virtual reality environment, with their movement on a spherical treadmill controlling motion in the environment. This closed-loop coupling between the animal’s motion and its perceptual experience were crucial to the generation of mismatch signals (Keller et al., 2012; Vasilevskaya et al., 2022).

A more recent paper questioned these results, however, arguing that mismatch signals could be explained by canonical V1 response properties such as locomotion gain, and orientation or direction selectivity (Muzzu and Saleem, 2021). The authors presented mice with visual-flow-mimicking drifting gratings that randomly halted. A subset of neurons responded robustly to the perturbations. Responses were enhanced by locomotion and congruent with the neurons’ orientation selectivity. Muzzu and Saleem also reasoned that a mouse’s tendency to move forward would, under efficient coding, establish a preference for front-to-back optic flow, but they found no such preference. While this result is interesting and suggestive of further experimentation, it is not necessarily conclusive. Most importantly, the experiment was performed in open loop with drifting gratings, quite distinct from closed-loop natural visual flow inputs. Predictive and efficient coding models predict that violations of expected visual flow will generate mismatch or error signals, based on the statistics of the *natural environment*. It is very difficult to know how the system ought to respond to non-natural visual flow inputs, especially when those are decoupled from the animal’s movement. As argued in a recent rebuttal to the Muzzu and Saleem paper from the Keller lab (Vasilevskaya et al., 2022), closed-loop coupling between locomotion and visual flow is crucial: responses to coupled perturbations (termed mismatches) were at least twice as large as responses during yoked open-loop perturbations (after controlling for locomotion speed). This difference between closed- and open-loop perturbations was absent in mice raised in an environment with no visuomotor coupling (Attinger et al., 2017). Furthermore, visual inputs during complex behaviors, for example rearing and turns, occur in all directions and so a preference for front-to-back visual flow is not necessarily expected.

As this example suggests, tests of efficient and predictive coding should be performed with stimuli matched to the animal’s natural environment, for example with a mouse freely exploring a nest or grassy field, or after sufficient training for the system to have learned the statistics of an unnatural environment (assuming such learning is possible). Indeed, a recent large-scale survey of neural activity across the mouse visual system showed substantial differences in neural tuning properties and overall activity in response to different types of visual input (De Vries et al., 2020). Many cells were unresponsive to entire classes of visual stimuli, such as natural scenes, while responding robustly to other classes, like drifting gratings. A related example comes from a study in Michael Stryker’s lab (Dyballa et al., 2018). Dyballa et al. analyzed the responses of V1 neurons to flow-like videos designed to imitate a mouse’s motion through grass and found robust visual responsiveness to spatial frequencies as high as about 1.5 cycles per degree, significantly greater than traditional visual acuity estimates of ∼0.5 cycles per degree measured using sinusoidal gratings (Porciatti et al., 1999; Prusky et al., 2000; Niell and Stryker, 2008). This difference may represent an in-vivo demonstration of the principle that codes are efficient only for the statistical environment to which they are matched. For future studies of efficient coding, experiments like those performed in the Keller and Stryker labs may provide a good compromise between experimental tractability and naturalistic stimuli/behavior.

### Sequential Visual Data

Real-world information streams exhibit both ordinal and temporal statistical dependences. A dancer might observe the continuous sequence of body movements required to perform a routine, or a driver might learn the discrete order and timing of turns along a route. Natural data streams contain both order and precise timing information. Any accurate model of these streams must describe *how often* different elements occur, *what order* they follow, and *when* they occur relative to each other. If the resulting codes are efficient, unexpected stimuli should evoke excess activity, or prediction errors, relative to expected stimuli. Depending on the nature of the encoding, prediction errors could be elicited by unexpected elements introduced to a sequence, expected elements rearranged within the sequence or omitted altogether, or expected elements presented at unexpected times. The literature contains a variety of terms describing these effects, including surprise-related enhancement, mismatch negativity, and prediction error. Ideally, responses would scale as the log of event probability, −log[*P*(*X*)] (Shannon, 1948; Cover and Thomas, 2006a; Stone, 2018), though this will ultimately depend on the details of the nervous system’s model of its natural environment and the ability of a non-negative, discrete signal (spikes) to encode probabilities. Note that contrived experimental sequential stimuli may evoke prediction errors, but only if the system has previously learned the frequency, order, and/or timing of those sequences.

Experimental sequences of discrete stimuli are usually designed to be predictable, sometimes obeying a Markov chain, and have provided evidence to support efficient coding models. Vinken et al. (2017) recorded single-unit responses in rat V1 and latero-intermediate area (LI) to random sequences of standard (90%) and oddball (10%) images. In this experiment, sequence order was irrelevant, so the thing to be learned was element frequency. In both areas, responses to standard elements were suppressed in a manner consistent with known adaptation mechanisms, but oddball elements drove significantly greater responses than control elements only in area LI. This effect was difficult to explain through adaptation. No such oddball response was observed in a similar experiment performed in monkey inferotemporal (IT) cortex (Kaliukhovich and Vogels, 2014). However, both studies exposed animals to sequences only during individual recording sessions. When monkeys were passively exposed to sequences containing ordinal information for much longer periods of time, there was evidence in IT for an enhanced response to order-violating stimuli (Meyer and Olson, 2011; Muckli et al., 2020). In other sensory modalities, especially audition, similar effects have been observed (Rubin et al., 2016; Heilbron and Chait, 2018; Maheu et al., 2019; Denham and Winkler, 2020). Given the more strictly temporal nature of auditory information, models in that modality may provide a source of inspiration for studies of visual temporal processing.

Many related studies have shown evidence for cortical novelty responses across brain regions, with wide variation in the effort to control for adaptation (Kato et al., 2015; Makino and Komiyama, 2015; Garrett et al., 2020; Poort et al., 2021; Schulz et al., 2021; Homann et al., 2022; Montgomery et al., 2022). In most cases, consistent with efficient coding, novel stimuli evoke more spiking activity than familiar. However, some studies have reported that familiar drive larger responses than novel stimuli. An example of this was seen in V1, where Gavornik and Bear (2014) repeatedly presented mice with a sequence of rapidly flashed sinusoidal gratings. Over a learning period of 5 days, the magnitude of visually evoked potentials increased dramatically in response to the trained sequence. Sequences violating trained expectations (including novel order, novel timing, and omitted elements) elicited responses that could be interpreted as error signals, but these were smaller than responses to the trained sequence. This result could reflect the fact that local field potentials largely represent synaptic currents in the dendrites (e.g., inputs) rather than neural spiking (e.g., outputs, Katzner et al., 2009). In more recent experiments in our lab, we have found that expectation-violating stimuli tend to elicit more spiking activity (Price et al., 2022). In addition to this unsupervised learning paradigm, there is also evidence for timing information in V1 following reinforcement learning (Shuler and Bear, 2006; Hangya and Kepecs, 2015; Levy et al., 2017). Interestingly, there is evidence that both the Gavornik and Bear sequence learning and Shuler and Bear reward timing paradigms (Chubykin et al., 2013) require cholinergic signaling, suggesting that this neurotransmitter may be uniquely required for plasticity that encodes temporal expectations into cortical circuits.

Other studies have found evidence for ordinal or temporal information in V1 using continuous-time stimuli (rather than discrete as above). One recent series of papers on “perceptual straightening” are particularly relevant to addressing whether the cortex produces efficient codes of spatiotemporal information (Hénaff et al., 2019, 2021). The authors argue that prediction is a fundamental cortical computation, and that it is easier to make predictions in V1 if the complex pattern of spike trajectories generated by the retina are “straightened” so that they evolve according to more-nearly linear dynamics. They find evidence for straightening in both human psychophysics experiments and macaque V1 (though the monkey data was recorded under anesthesia). Another study found that navigation within a virtual environment creates responses in mouse V1 that are increasingly predictive of upcoming stimuli, such that omissions of expected stimuli drive high activity (Fiser et al., 2016). This work ties into recent evidence for a strong functional relationship between V1 and hippocampus in the mouse, with V1 showing spatial modulation in virtual environments consistent with the hippocampal representation of space (Saleem et al., 2018; Diamanti et al., 2021). Interestingly, the Gavornik and Bear result was recently shown to require an intact hippocampus for plasticity induction (Finnie et al., 2021). Overall, these results blur the distinction between visual coding and memory and illustrate how difficult it is to establish an experimental paradigm to test the efficient coding hypothesis in cortex and based on visual inputs alone.

### Adaptation

Adaptation describes the time-varying behavior of neurons as they adjust their firing properties to changes in environmental statistics. A classic example is the change in dynamic range of retinal photoreceptors in response to changes in overall light intensity (Normann and Perlman, 1979; Carandini and Heeger, 2012). After adapting to a dark environment, photoreceptor responses saturate at daytime light intensities and are thereby rendered temporarily unable to transmit information at these higher intensities (Normann and Perlman, 1979). This is precisely the behavior predicted by efficient coding, since it allows neurons to maximize information throughput under changing conditions. Comparable effects have also been observed in blowfly H1 neurons under a variety of experimental conditions, where adaptation provably maximizes information transmission (Brenner et al., 2000; Fairhall et al., 2001). Adaptation, at least in the very early visual system, can therefore be understood as a consequence of efficient coding principles (Barlow and Földiák, 1989; Meister and Berry, 1999; Wainwright, 1999; Weber et al., 2019).

Natural scenes are non-stationary and dynamic at various timescales. In context of this review, we are primarily interested in how patterns in the temporal domain create probabilistic dependences between different moments in time and how these can be used to make predictions. Efficient coding suggests neural circuits should learn these dependences and remove them from the neural code, creating time-invariant representations of objects and other environmental features and allowing for prediction of future states. In this framing, adaptation can be a confound to tests of efficient or predictive coding.

**FIGURE 3:**
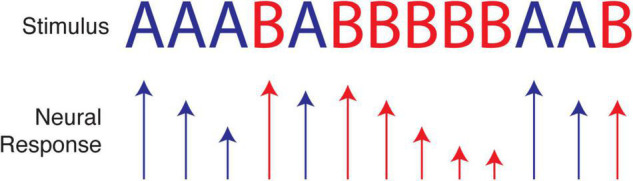
Neural adaptation caused by repetition of a single stimulus over a short period of time can cause neural responses to decrease in magnitude. Relatively large responses, as when A follows BBBBB, can be interpreted as signifying either a prediction violation or a simple lack of adaption in the population of neurons selective for A. Depending on the input statistics, these responses could be efficient, as expected stimuli (assuming we expect repeats) are represented with less activity than unexpected stimuli. For this reason, it is not always clear if adaptation is a confound to studying efficient temporal coding, a mechanism implementing it, or some mixture of the two.

As a simple illustration, consider an experiment that presents a sequence of two visual stimuli where each element presentation is separated by 100 ms, for example AAABABBBBBAAB. Due to synaptic depression and other known forms of adaptation (Carandini and Ferster, 1997; Chung et al., 2002; Blitz et al., 2004), cortical responses to one particular stimulus will often decrease with repeated presentations, e.g., BBBBB. The response to A following this run will be greater not only than the last response to B but also than the average response to A (which includes diminished responses from runs of A’s). Paired-pulse-like facilitation may also occur for patterns like AA or ABA, creating even more complex response profiles. The wide range of adaptation-like mechanisms observed in neural tissue therefore present an obvious challenge to tests of efficient and predictive coding, because the former do not seem to require knowledge of an underlying probability distribution. At the same time, we know adaptation can serve the principle of efficient coding, as described above and outlined in an excellent review (Weber et al., 2019). Adaptation may therefore provide a mechanistic implementation of efficient coding for certain stimulus distributions, without the need for any form of long-term plasticity to encode temporal relationships (see Figure 3). This may be demonstrated by a recent paper showing that novel stimuli presented within repeatable sequences evoke excess activity, as predicted by efficient coding (Homann et al., 2022). The proposed mechanism, however, was consistent with a straightforward adaptation model. In certain cases, it is possible to dissociate predictive coding from adaptation (Tang et al., 2018), though not all adaptive mechanisms are known or understood, and empirical predictions for predictive and efficient coding in cortical circuitry are not as well developed as they have been in the retina and dLGN.

## Criticisms

Though efficient coding and information theory are clearly relevant to neural computation, there is much debate regarding the extent to which these ideas explain what we see in the nervous system, especially beyond the retina and dLGN. We will walk through a point-counterpoint debate that emphasizes three prominent criticisms of efficient and predictive coding as theories of cortical function (see Simoncelli, 2003 for a complementary perspective). (1) The massive expansion in the number of neurons from dLGN to V1 would appear to increase redundancy, contrary to the efficient coding hypothesis. (2) There is functional evidence that contradicts predictive coding theory. (3) Efficient coding makes very precise, testable predictions in certain contexts, but, in general, information-theoretic measures are very difficult, if not impossible, to estimate (Paninski, 2003) making the overall utility of applying efficient coding to the cortex unclear.

The first criticism is suggestive of the inherent difficulty in translating information-theoretic ideas to the nervous system. Unlike a telephone system, where engineers need only concern themselves with transferring data efficiently, cortical circuits must both encode information and operate on it. For computer hard drives, efficiency is defined by minimizing the average number of bits utilized while preserving information from known sources of noise. By comparison, efficiency in the nervous system might mean minimizing the average number of spikes per second while preserving information, or maximizing the average number of bits per molecule of ATP, or maximizing bits per volume of axon (Laughlin et al., 1998; Balasubramanian and Sterling, 2009; Sterling and Laughlin, 2015; Stone, 2018). In the retina, Balasubramanian and Sterling (2009) proposed the following instantiation of efficient coding theory: “Given the information required for behavior, the retina minimizes its computational costs.” By precisely measuring the metabolic and computational costs of information processing under certain conditions, these researchers and others (Laughlin et al., 1998) have found that each additional bit of information transmitted along the optic nerve requires correspondingly more neural resources, space, and energy, creating a dramatic law of diminishing returns (Perge et al., 2009). This explains many of the functional properties of the retina, including its differentiation into multiple parallel processing streams (Borghuis et al., 2008; Balasubramanian and Sterling, 2009; Gjorgjieva et al., 2014; Sterling and Laughlin, 2015; Ocko et al., 2018). Overall, such considerations reflect the subtlety of the problem and the need for very precise specifications of the theory. In V1, the fact that we find more neurons than in dLGN does constitute an increase in physical resources such as space and protein molecules, but it could well cause a decrement in the average number of spikes or in the redundancy of messages transmitted beyond V1. The expansion might also reflect a requirement for additional neural resources in the cortex as V1 integrates information across multiple modalities, or reflect cells that are being used for other cortical functions.

Regarding the second criticism, there are many examples in the literature that seem to contradict efficient and predictive coding. One such example comes from a study by Benucci et al. (2009), where the authors studied neural responses to sequences of oriented gratings in anesthetized cat visual cortex. Membrane potentials measured in response to the sequences were highly predictable from a simple linear model of the responses to individual gratings. Thus, the temporal context in which the gratings were displayed was irrelevant. The authors therefore concluded “spatial and temporal codes in area V1 operate largely independently.” A more recent study by Solomon et al. looked at neural responses in awake macaque and human visual cortex in response to rapidly flashed sequences of sinusoidal gratings (Solomon et al., 2021). They showed *standard* sequences on 80% of trials and *deviant* sequences on 20%, expecting to observe prediction errors in response to deviant stimuli. Instead, they found minimal evidence for prediction errors, with the responses to deviant and standard stimuli being almost identical.

Both studies establish crucial limitations to efficient and predictive coding, but do not invalidate the theories. Importantly, the studies presented sequences of non-natural sinusoidal grating stimuli within individual recording sessions, leaving little time for the neural system to learn the new statistics. Efficient and predictive coding both suggest that expectations are either evolved or learned relative to the natural visual environment, so a random sequence of sinusoidal gratings is always novel/unexpected from that perspective (regardless of whether it came from a standard or deviant set). There is no *a priori* reason to expect the visual system would learn to differentiate standard from deviant stimuli within a recording session. The Solomon et al. result therefore might suggest a limitation to predictive coding: monkeys and humans do not seem to learn non-natural sequences of stimuli on a timescale of minutes to hours (though see also Ekman et al., 2017, which demonstrated anticipatory cue-evoked pre-play of expected visual trajectories in human V1 after a brief period of training). Given more exposure time, they may or may not learn such sequences, depending on the ability of the visual system to flexibly adapt and modify its internal expectations. The use of anesthetized cats in Benucci et al. result is particularly problematic from an interpretive standpoint, since anesthesia seems to be preferentially disrupt cortical processing (Voss et al., 2019). The absence of contextual modulation may reveal little about how the awake brain exploits temporal relationships to make predictions.

The third criticism is perhaps the most difficult, as demonstrated by a simple thought experiment. Suppose we hypothesize that V1 compresses information arriving from dLGN before sending it to V2 and that we want to test this hypothesis in the spatial domain. We might devise an experiment to measure the entropy of dLGN and V1 projection neurons in response to natural scenes. The summed entropies of all V1-projecting dLGN neurons is the average “message length” of that population, likewise for the summed entropies of V1 neurons transmitting to V2. Formally, our hypothesis would be:


$$\sum_{i=1}^{N}H\left(L_{i}\right)>\sum_{j=1}^{M}H\left(V_{j}\right)\;I\left(S;L\right)\approx I\left(S;V\right)$$

where *H*(*L*_*i*_) is the entropy of the i-th dLGN projection neuron (of *N* total), *H*(*V*_*j*_) is the entropy of the j-th V1 projection neuron, and *I*(*S*; *L*) is the mutual information between sensory inputs and the population response in dLGN. Input information is preserved at the output of V1 but in a compressed form. In theory, we would need to record from a very large number of neurons for a very long time to test this hypothesis. Accurate estimation of the individual entropies is tractable under certain assumptions, but estimation of the mutual information would be impossible in any realistic neuroscience experiment due to the curse of dimensionality (Paninski, 2003). Were we to include the temporal domain as well, by showing natural movies for example, neural responses at different timepoints would no longer be independent. Entropies and mutual information become exponentially more difficult to estimate. When you begin to consider the complexity of the circuit, rife with feedback and interconnections, the problem becomes even more difficult to specify. Therefore, while it is fairly straightforward to devise hypotheses around efficient coding, it is difficult to see how they will ever be tested. This is a fair criticism, but it is not unique to this specific theory: there is rarely an easy way to compare neural data to theory. Further, there are many ways to test ideas of efficient coding. Most are inconclusive or incomplete individually, but cumulative evidence supporting the theory can still accumulate over time.

## Discussion

One of the things that makes it so difficult to understand the brain is the mechanistic overlap between computation and representation. The conscious percept of a particular thought or idea or image somehow emerges from the combined activity of populations of neurons, and it is probably correct to say that the population activity defines the neural code representing the idea. This same population, however, participates in the input-output transformations responsible for computation. In a real sense, computation and representation are inseparable aspects of neural activity and there are interpretive dangers in focusing exclusively on either. Given these challenges, it is natural to question the extent to which a mathematical framework developed to help optimize data transfer and storage in engineered telecommunication systems can provide insight into brain function. This review has highlighted some of the difficulties in applying information theory to the visual cortex, and it seems unlikely that this (or perhaps any) theory will fully explain the brain’s complex neurobiology.

That said, information theory provides a useful framework to understand how evolutionary pressure toward efficient resource utilization can create predictive coding schemes with an intrinsic role for time. The complex, spatiotemporal distribution of visual information means that if the brain uses an efficient coding strategy anywhere, visual areas are an ideal candidate. Natural visual scenes exhibit autocorrelations that are useful for implying causality and predicting the future or reconstructing the past. A key insight is that a drive toward efficiency encourages temporal relationships to be represented in the neural code. Efficient coding theory also implies that there ought to be selective pressure to learn approximate space-time distributions over natural visual inputs and provides an account of how sensory data ought to be encoded. In particular, neuroscientists may expect to find evidence that data is compressed by removal of predictable spatial and temporal information, thus displaying a degree of spatial and temporal invariance.

The theory also implies that unexpected or unpredictable patterns ought to elicit error signals that would most likely be coded by *increased* firing rates at either the individual neuron or population level (e.g., an unexpected stimulus could also increase the size of the response population). Neurons in the retina, dLGN, and V1 all show functional properties consistent with this hypothesis (as reviewed above) and higher visual areas may be consistent with this theory as well (Jehee et al., 2006; Beyeler et al., 2016; Piasini et al., 2021). An important thing to note is that predictions are based on the environmental statistics responsible for creating the internal model, and it is not clear what to expect when the system is challenged by inputs with different statistics. This implies that experiments testing neural coding in the visual system should either use stimuli with naturalistic statistics or incorporate a period of training sufficient to encode new statistics into the neural circuits before looking for evidence of predictive processing.

Based on the current state of the field, there are many open questions for future research. In our opinion, one of the first steps should be to more fully characterize the visual system’s model of the visual environment. To what extent does that model incorporate the temporal dimension? On what timescales? Does the system predict the sensory consequences of the animal’s behavior (i.e., is it a joint model of inputs and outputs)? To what extent is the model capable of incorporating new statistics? It is generally a good idea to dissociate characterizations of the modeled environmental distribution from determinations of whether that distribution is efficiently encoded (not least because there are multiple possible dimensions along which efficiency could be measured). Most experimental stimuli include spatiotemporal content that is distinct from the animal’s normal perceptual experience. If the visual system cares about both space and time, and is flexible enough to learn, then presentation of these novel stimuli ought to induce a learning process. Another important experimental goal is therefore to characterize the extent to which the visual system can learn novel distributions, the timescale over which this learning occurs, and the overlap with known plasticity mechanisms.

We have focused this review largely on work in the early rodent visual system, but there is a larger body of literature relevant to this discussion in other brain regions and model systems, and other experimental paradigms, that could be adapted to address the issue; for example, the long-standing hypothesis that “what” and “where” information are processed in parallel ventral and dorsal pathways in primates (Ungerleider and Mishkin, 1982; Goodale and Milner, 1992; Milner and Goodale, 2008). A similar division seems to exist in the mouse visual system as well (Marshel et al., 2011; Garrett et al., 2014; Bakhtiari et al., 2021; Siegle et al., 2021). This functional segregation of space-like and time-like pathways leads to the assumption that while time is explicitly required in the dorsal stream to process motion, in the ventral stream it is useful only to integrate over noisy sensory data. Consequently, many visual processing models work only on static images. This is especially true for object recognition but also models of efficient and predictive coding (Olshausen and Field, 1996; Bell and Sejnowski, 1997; Rao and Ballard, 1999; Riesenhuber and Poggio, 1999; Carandini et al., 2005; Yamins et al., 2014; Cadena et al., 2019; Sanchez-Giraldo et al., 2019). There are theoretical arguments, though, that time could be explicitly used for computations in the ventral pathway. For example, temporal information can be explicitly useful for object recognition (Stone, 1998, 1999), or to identify kinetic borders when camouflaged objects move through the visual environment (Cavanagh and Mather, 1989; Layton and Yazdanbakhsh, 2015). This suggests that experiments manipulating temporal expectation could be used in both the ventral and dorsal streams to determine the extent to which object recognition, localization, border assignment, etc. rely on efficient spatiotemporal coding principles. Given the approximate homogeneity of cortical circuits in visual and non-visual areas, it is likely that the principles used to encode visual information will be useful to understand general cortical processing algorithms as well.

## Author Contributions

BP and JG wrote the manuscript. Both authors contributed to the article and approved the submitted version.

## Conflict of Interest

The authors declare that the research was conducted in the absence of any commercial or financial relationships that could be construed as a potential conflict of interest.

## Publisher’s Note

All claims expressed in this article are solely those of the authors and do not necessarily represent those of their affiliated organizations, or those of the publisher, the editors and the reviewers. Any product that may be evaluated in this article, or claim that may be made by its manufacturer, is not guaranteed or endorsed by the publisher.
